# Asymmetric Spin Canting and Demagnetization Dynamics Driven by Laser Fields in Two-Dimensional Altermagnets

**DOI:** 10.1021/acs.nanolett.5c04244

**Published:** 2026-02-09

**Authors:** Shuo Li, Ran Wang, Thomas Frauenheim, Zhaobo Zhou, Junjie He

**Affiliations:** † Institute for Advanced Study, 74707 Chengdu University, Chengdu 610106, China; ‡ School of Science, Constructor University, 28759 Bremen, Germany; § Faculty of Science, 37740 Charles University, 12843 Prague, Czech Republic

**Keywords:** two-dimensional altermagnets, asymmetric demagnetization dynamics, spin canting, a-OISTR, TDDFT

## Abstract

Laser-induced ultrafast magnetization dynamics have been well established in conventional magnets but remain unexplored in altermagnets (AMs). Using real-time time-dependent density functional theory (rt-TDDFT), we demonstrate that laser pulses can drive asymmetric demagnetization dynamics between the two Fe sublattices in the two-dimensional (2D) semiconducting AM, Fe_2_WTe_4_, leading to a photoinduced ferrimagnetic state with a net magnetization of approximately 0.3 μ_B_ per unit cell. This metastable magnetization originates from the momentum-dependent spin-splitting characteristic of *d*-wave AMs, which gives rise to an anisotropic optical intersite spin transfer effect (OISTR). Furthermore, the asymmetric demagnetization is accompanied by non-collinear spin dynamics, resulting in distinct spin canting angles for two Fe sublattices. Importantly, these spin dynamics are tunable by the in-plane polarization angle of the laser field. Our findings provide microscopic insight into the ultrafast control of magnetization in 2D AMs and open new avenues for light-driven manipulation of spin textures in AM systems.

## Graphical abstract

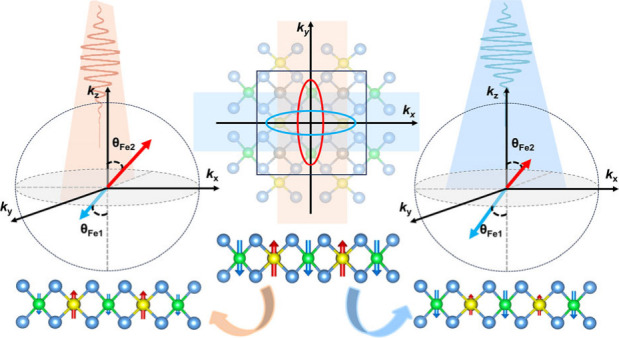

Magnetism has long been characterized by two distinct phases: ferromagnets (FMs) with net magnetization and antiferromagnets (AFMs) with compensated spins.^1–4^ FMs facilitate spin-polarized transport; however, their stray fields and GHz-limited dynamics impede high-density integration.^1,2^ In contrast, AFMs demonstrate the phenomenon of terahertz resonance and exhibit immunity to stray fields. However, they are characterized by uncontrollable order and weak signals.^4–6^ This dichotomy has been overcome by the recent discovery of altermagnets (AMs), which are defined as a compensated magnetic phase with vanishing net magnetization yet robust time-reversal symmetry breaking.^7,8^ As a consequence of non-relativistic alternating spin splitting (e.g., *d*/*g*/*i*-wave symmetry in momentum space), AMs exhibit momentum-locked spin polarization devoid of spin–orbit coupling.^7^ These dual-phase characteristics (e.g., AFM-like compensation and FM-like band splitting) facilitate unprecedented phenomena, including a giant anomalous Hall effect^8^ and non-relativistic spin currents,^9^ positioning AMs as a revolutionary platform for high-speed, low-power spintronics.

The candidates for two-dimensional (2D) AMs are less than those for their bulk counterparts due to the more stringent symmetry requirements.^10^ Layered materials, like the CrSb thin films^11^ and the room-temperature metallic Rb_1−δ_V_2_Te_2_O compounds,^12^ bridge three-dimensional (3D) and 2D regimes, exhibiting preserved spin-split bands down to atomic thicknesses. 2D AMs have been demonstrated to offer tunability of spin orientation in *k* space, such as electric-field-induced AM transitions,^13^ Janus structural control,^14,15^ and stack/twist-engineered band splitting.^16,17^ The sensitivity exhibited by 2D AMs is attributed to the breaking of the time-reversal and spatial inversion symmetries.^8^ Nevertheless, the interactions between light and 2D AM remain largely unexplored for magnetic control toward attosecond/femtosecond time scales.

Ultrafast magnetization dynamics, first observed in Ni by Beaurepaire et al. in 1996, has since been widely explored in conventional ferromagnetic and antiferromagnetic systems under laser excitation.^18^ In particular, the optical-induced intersite spin transfer (OISTR) as a key theoretical breakthrough has been demonstrated in conventional magnets,^19–23^ revealing that ultrafast laser pulses can redistribute spins across atomic sites in magnetic systems.^24^ More recently, theoretical predictions and early experimental efforts have suggested that the ultrafast spin dynamics and optical responses in AMs may exhibit fundamentally different behavior from those in conventional magnets.^25–33^ This distinction arises from the unique nodal structures in momentum space. Particularly, Zhou et al.^25,33^ demonstrated that the momentum-dependent spin splitting in AMs enables an anisotropic optical-induced intersite spin transfer (a-OISTR) effect under linearly polarized light. This a-OISTR mechanism drives asymmetric demagnetization between the two compensated sublattices, leading to the ultrafast generation of a photoinduced metastable ferrimagnetic state with a net magnetic moment, as shown in 3D *d*-wave (RuO_2_) and *g*-wave (CrSb) prototypes. The general principle of a-OISTR is that asymmetric/symmetric local density of states gives rise to asymmetric/symmetric demagnetization dynamics. Consequently, the spin dynamics and polarization-dependent OISTR response are critically governed by the band-path-resolved local density of states. Recently, some experimental studies have probed AMs under laser excitation using techniques, such as the time-resolved magneto-optical Kerr effect,^34,35^ angle-resolved photoemission spectroscopy,^36–38^ and magnetic circular dichroism.^39^ Despite these advances, the study of laser-induced ultrafast magnetization dynamics in AMs remains at a very early stage. However, these studies focused on 3D bulk AMs. The a-OISTR in low-dimensional AM systems remains unclear. Reduced symmetry and different magnetic anisotropies could alter ultrafast spin dynamics. The emergence of 2D AMs provides an opportunity to investigate these effects at the ultimate limit of thinness.

In this work, using real-time time-dependent density functional theory (rt-TDDFT), we systematically investigate the femtosecond spin dynamics under a varying laser field in a prototypical 2D semiconducting *d*-wave AM, Fe_2_WTe_4_.^40^ We show that laser pulses induce a pronounced asymmetry in the spin dynamics between sublattices, resulting in a photoinduced ferrimagnetic state with a sizable net magnetization and spin canting. This emergent magnetization is strongly dependent on the in-plane polarization angle of the laser field. The origin of this ultrafast ferrimagnetic state lies in the a-OISTR, which is attributed to the nodal electronic structure inherent to *d*-wave AM. Overall, our findings uncover a microscopic mechanism for laser-induced spin dynamics in 2D altermagnets.

The optimized structure of Fe_2_WTe_4_ with the Te–Fe–Te sandwich structure is shown in Figure 1a, which possesses the space group of *P*4̅2*m*. The crystal arrangement of W and Te atoms breaks the *PT* symmetry of magnetization density on the opposite Fe spin sublattices.^40,41^ Therefore, Fe_2_WTe_4_ is regarded as a type-I altermagnet,^42^ where its spin splitting is independent of spin–orbital coupling (SOC). Figure 1c shows the band structure of Fe_2_WTe_4_, resulting in pronounced spin splitting along the M–X−Γ–Y–M paths, while spin degeneracy is observed along the Γ–M path. Moreover, the band structures of Fe_2_WTe_4_ at different *U* values are shown in Figure S1. Furthermore, Figure 1d shows the projected band structure of Fe_1_ and Fe_2_ atoms, in which two valence band maxima (VBM) in spin-down and spin-up channels exhibit at *X* and *Y* valleys.

**Figure 1:**
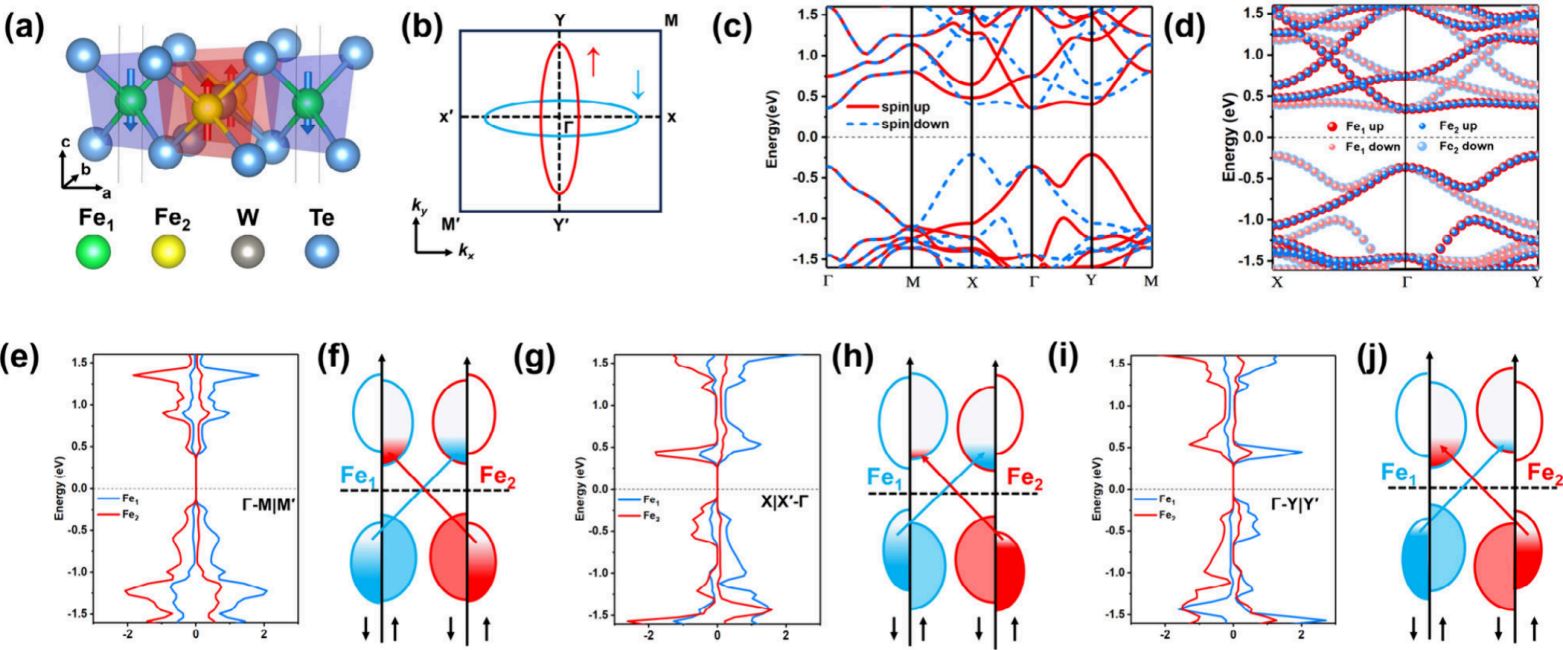
(a) Crystal structure of Fe_2_WTe_4_ with two spin sublattices. Green, yellow, gray, and blue spheres represent Fe_1_, Fe_2_, W, and Te atoms, respectively. Blue and red arrows represent the opposite Néel vectors of two Fe atoms in the unit cell. (b) 2D Brillouin zone of Fe_2_WTe_4_. Blue and red colors highlight the alternating symmetry of spin polarization, corresponding to the spin-down and spin-up states of two spin sublattices, respectively. (c) Band structure of Fe_2_WTe_4_ without SOC. (d) Projected band structure of Fe_1_ and Fe_2_ atoms in FeFe_2_WTe_4_ without SOC. (e, g, and i) Spin-resolved density of states of Fe_1_ and Fe_2_ atoms along Γ–M|M′, X|X′−Γ, and Γ–Y|Y′ paths. (f, h, and j) Schematic of an a-OISTR process in Fe_2_WTe_4_. Red and blue arrows represent the spin transfer in two spin sublattices along different high-symmetry paths.

The unique band structure of the 2D AM could result in a variety of light-induced spin responses. This also distinguishes it from conventional AFMs. Therefore, the new mechanism of spin dynamics in AM is worthy of an in-depth exploration. For AFMs, the absence of spin splitting in the ground-state bands ensures symmetric charge flow between the majority and minority spin channels when they are pumped by a laser. This results in symmetric demagnetization across both sublattices. For Fe_2_WTe_4_, its symmetry is broken along the direction-dependent spin polarization (Figure 1e, g, and i), resulting in ana-OISTR. Due to the different band gaps in the spin-up and spin-down channels, i.e., different band edge positions, this will result in a non-compensated spin transfer process. For example, along X|X′−Γ paths, the spin-selective charges are more excited to the conduction band minimum (CBM) of Fe_2_ in the spin-down channel (Figure 1h). While along Γ–Y|Y′ paths, the spin-selective charges are more excited to the VBM of Fe_1_ in the spin-up channel (Figure 1j). This asymmetric excitation of spin-selective charges gives rise to an asymmetric demagnetization process. Therefore, a phase transition from AFM to ferrimagnetic occurs, resulting in a laser-induced spin dynamics process of the net magnetic moment. Furthermore, in the case of the symmetric band edge positions, the spin-selective charge excitation process remains degenerate (Figure 1f), and the demagnetization process is analogous to that of AFM. Furthermore, the indirect excitation pathways could exist,^43^ such as in indirect spin transfer involving the non-magnetic W and Te atoms through their orbital hybridization. However, the primary channel for the a-OISTR effect is spin transfer between the two Fe sublattices.

Based on the above understanding of the a-OISTR, we will demonstrate this physical phenomenon in Fe_2_WTe_4_ by using the *ab initio* rt-TDDFT simulations. The in-plane polarization angle is defined as the angle between the electric field (**E** vector) of the laser pulse and the *k*_*x*_ axis (Figure 2a). The laser pulse will be rotated; i.e., the polarization angle α will be equal to 0°, 45°, and 90°, respectively. The **E** vector of the laser pulse is chosen parallel to the directions X−Γ–X′, M−Γ–M′, and Y−Γ–Y′, respectively, for α = 0°, 45°, and 90°. The laser pulse irradiation in the direction of α = 0°/90° and 45° will be referred to as the direction parallel to the spin-polarized and spin-degenerate paths. Moreover, the vector potential of the laser pulse is shown in Figure 2b.

**Figure 2:**
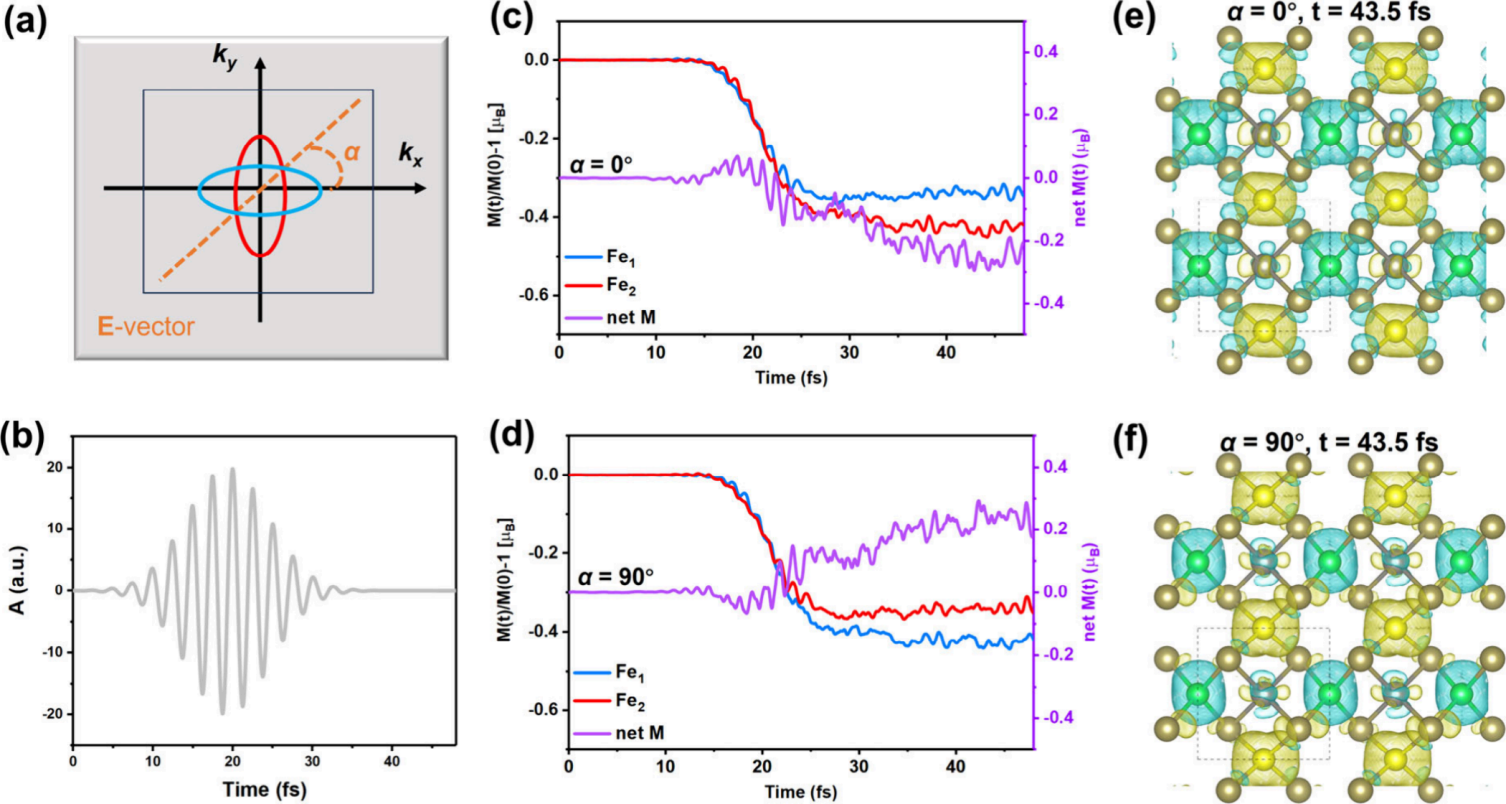
(a) Schematic of Fe_2_WTe_4_ under laser pulse irradiation with polarization angle α, which is defined as the angle between the electric field vector (**E** vector) of the laser and the *k*_*x*_ axis. (b) Vector potential of the laser pulse (frequency, *f* = 1.63 eV; full width at half maximum, fwhm = ∼10 fs; and fluence, *F* = 12.29 mJ/cm^2^). (c and d) Normalized Fe atom-resolved spin moment as a function of time at α = 0° and 90°, respectively. The net magnetic moment (net M) is shown in purple. (e and f) Magnetization density of Fe_2_WTe_4_ at α = 0° and 90°, respectively. Yellow and green domains indicate the spin-up and spin-down magnetization density, respectively. The isosurface is set to 0.0012 *e*/Bohr^3^.

We simulated the demagnetization of Fe atoms excited by the laser pulses. The normalized spin moments of Fe atoms over time for two polarization angles are demonstrated in Figure 2c and d. Along the spin-polarized paths at α = 0° and 90°, Fe_1_ and Fe_2_ atoms exhibit unequal demagnetization, resulting in ferrimagnetic polarization with a net moment of approximately up to 0.3 μ_B_ within 45 fs. The corresponding magnetization density of Fe_2_WTe_4_ at α = 0° and 90° is shown in Figure 2e and f. Moreover, the Fe_2_ atom exhibits a larger spin moment loss at 0° than the Fe_1_ atom, whereas the opposite trend is observed at α = 90°. When α = 0°, the **E** vector of the laser is parallel to the X−Γ–X′ path. The spin-selective charges are more excited from the VBM of Fe_1_ to the CBM of Fe_2_ in the spin-down channel than from the VBM of Fe_2_ to the CBM of Fe_1_ in the spin-up channel, resulting in the increasing demagnetization of the Fe_2_ atom. Additionally, the influence of laser parameters on the demagnetization of Fe atoms is shown in Figure S2. The converse case will be observed when angle α is equal to 90°. This is all due to the a-OISTR effect in Fe_2_WTe_4_. Moreover, when α = 45°, the **E** vector of the laser is parallel to the M−Γ–M′ path and Fe_1_ and Fe_2_ atoms keep the symmetric demagnetization process with spin moment loss (Figure S3), similar to the conventional AFMs.

To further understand the role of the charge excitation in the spin dynamics, we calculated the change in time-resolved DOS [ΔDOS­(*t*)] for Fe_1_ and Fe_2_ atoms. This is defined as the difference between the DOS at time *t* = 43.5 and 0 fs, as shown in Figure 3. The results demonstrate a distinctly asymmetric charge accumulation process, in which electrons mainly populate the CBM of the Fe_1_ atom in the spin-up channel and the Fe_2_ atom in the spin-down channel. Therefore, this asymmetric demagnetization in Fe_2_WTe_4_ gives rise to a net magnetic moment of the Fe sublattices. Moreover, the electron loss of the Fe_2_ atom in the spin-down channel is greater than that of the Fe_1_ atom in the spin-up channel when α = 0°, while the reverse is true when α = 90° (Figure 3a and b). Notably, the broad energy range of the occupation changes arises from nonlinear light–matter interactions under the high fluence of the laser pulse,^44^ which can accelerate electrons to kinetic energies exceeding the photon energy of 1.63 eV. Consequently, the asymmetric spin dynamics resulting from direction-dependent spin-selective charge transfer directly correlates with stronger demagnetization, as evidenced by the observation of a spin-dependent asymmetric current flow.

**Figure 3:**
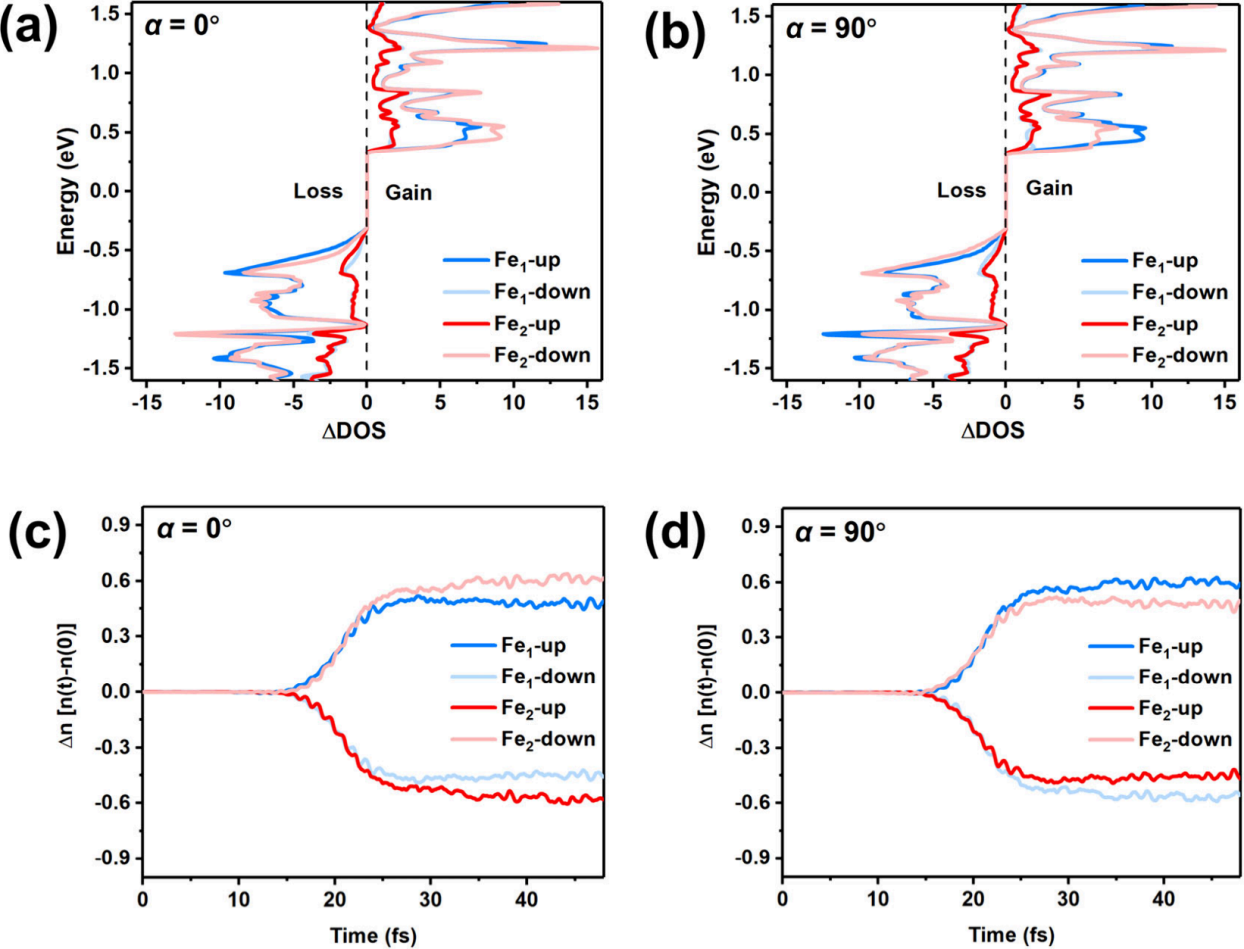
(a and b) Differences in the time-resolved occupation function ΔDOS­(*t*) at *t* = 43.5 fs. The negative value signifies a loss of electrons, and a positive value signifies a gain of electrons. (c and d) Change in the spin-resolved charges Δ*n* of two Fe atoms. The positive/negative value represents the increase/decrease of charges.

Furthermore, the time-dependent changes in the spin-resolved charge Δ*n* of Fe_1_ and Fe_2_ atoms are shown in Figure 3c and d, respectively. The utilization of spin-resolved charge dynamics has been identified as a means of characterizing alterations in spin moment loss, which is expressed as follows:

$$\Delta n_{↑}\left(t\right)=\left[\Delta n\left(t\right)+\Delta M\left(t\right)\right]/2$$

$$\Delta n_{↓}\left(t\right)=\left[\Delta n\left(t\right)-\Delta M\left(t\right)\right]/2$$

where Δ*n*_↑_(*t*) = *n*(*t*) – *n*(*t = 0*) represents the change in local charge compared to the initial charge. Δ*n*_↑_(*t*) and Δ*n*_↓_(*t*) denote the time-dependent changes in spin-up and spin-down charges, respectively. The change in spin moment can be defined as Δ*M*(*t*) = Δ*n*_↑_(*t*) – Δ*n*_↓_(*t*), where the higher the difference between Δ*n*_↑_(*t*) and Δ*n*_↓_(*t*), the more significant the loss in spin moment. The results demonstrate that Δ*n*_↑_(*t*) and Δ*n*_↓_(*t*) of Fe atoms exhibit an increase and decrease, suggesting the demagnetization process of Fe atoms in Fe_2_WTe_4_. Moreover, asymmetric Δ*n* of Fe_1_ and Fe_2_ is observed when the laser is directed along the spin-polarized paths, i.e., α = 0°/90°. Consequently, the asymmetric redistribution of the spin-resolved charge between Fe_1_ and Fe_2_ further corroborates a-OISTR and generation of ferrimagnetic polarization in Fe_2_WTe_4_. Additionally, when α = 45°, ΔDOS­(*t*) and Δ*n* of Fe_1_ and Fe_2_ atoms are symmetric (Figure S4), similar to the conventional AFMs.

We next investigate the non-collinear spin dynamics that emerge following asymmetric demagnetization. The spin-resolved band structures of Fe_2_WTe_4_ projected on the Fe *d* orbitals are shown in Figure S5. The results show the partial overlap between the S_*x*_/S_*y*_ band of Fe *d* orbitals and the S_*z*_ band along the X−Γ/Γ–Y paths. The spin orientation can be changed by the spin–orbit torque due to spin-momentum locking.^45^ Therefore, the laser-induced redistribution of electrons between the S_*x*_/S_*y*_ and S_*z*_ states would result in a change in the spin orientations of Fe atoms from M_*z*_ to M_*x*_/M_*y*_. Our results reveal that, during the demagnetization process in Fe_2_WTe_4_, the spin orientations of the Fe sublattices undergo noticeable reorientation. To quantify this behavior, we define the spin canting angle (θ) as the angle between the spin axis and the ±M_*z*_ axis (Figure 4a). The time evolution of the spin canting angle for Fe_1_ (θ_Fe_1__) and Fe_2_ (θ_Fe_2__) atoms under laser polarization angles at α = 0° and 90° is shown in Figure 4b and c. Under these conditions, non-collinear spin dynamics are clearly asymmetric: θ_Fe_1__ and θ_Fe_2__ exhibit different magnitudes, particularly at α = 90°, where the canting angle difference reaches up to approximately 30° within 75 fs (Figure 4c). We observed that the spin canting angle and the M_*x*_/M_*y*_ components continue to increase by approximately 55 fs. After that, the spins begin to oscillate and gradually approach a relatively stable state. This asymmetry reflects a strong sublattice-selective response under linearly polarized light. In contrast, at α = 45°, the spin canting angles of Fe_1_ and Fe_2_ increase by exactly the same amount, exhibiting a fully symmetric non-collinear response (Figure S6). This behavior is consistent with the observed symmetric demagnetization along the out-of-plane (*z*) direction at the same polarization angle, indicating a polarization-controlled crossover from asymmetric to symmetric spin dynamics. Moreover, the spin orientations of the *x* and *y* components are opposite at α = 0° and 90°, as demonstrated on the M_*x*_–M_*y*_ plane (Figure 4e and g). Moreover, the change in *x*, *y*, and *z* components of spin moment of Fe atoms at α = 0° and 90° is shown in Figure S7. While at α = 45°, the increase of M_*x*_ and M_*y*_ of Fe atoms is basically the same (Figure S8). Additionally, Figures S9 and S10 show the spin moment dynamics of the *x* and *y* components of the Fe atoms at α = 0° and the corresponding spin canting angle for the SOC scaled by factors of 0.1 and 0.5. It is evident that a decrease in the SOC leads to a decrease in the M_*x*_/M_*y*_ component. The SOC as an effective internal magnetic field exerts a continuous torque on the non-collinear spins, further driving them away from their initial collinear alignment. These results demonstrate that the asymmetric spin orientations and non-collinear dynamics of the Fe sublattices in Fe_2_WTe_4_ can be flexibly and precisely manipulated by tuning the polarization of the laser field, offering a new degree of control over spin textures in 2D altermagnets.

**Figure 4:**
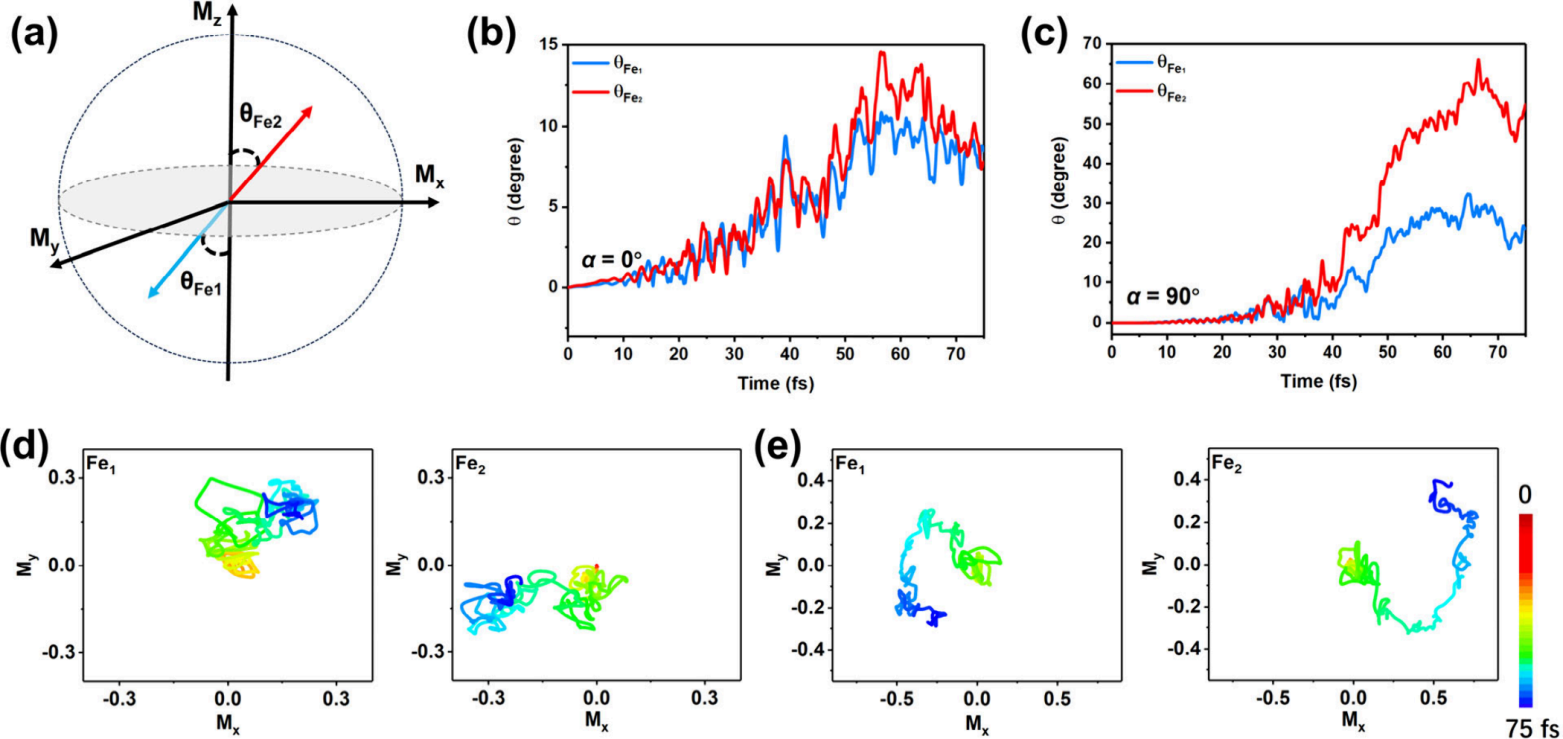
(a) Schematic diagram for the evolution of spin canting angle θ. (b and c) Spin canting angle θ as a function of time at α = 0° and 90°, respectively. (d and e) *x* and *y* components of spin moment of Fe atoms on the M_*x*_–M_*y*_ plane as a function of time at α = 0° and 90°, respectively. The color bar serves as the time scale.

The phenomenon of laser-induced ultrafast demagnetization has been investigated in femtomagnetism since its discovery in nickel,^18^ with OISTR emerging as a key mechanism driving femtosecond spin dynamics.^19^ However, a-OISTR in altermagnets is governed by a momentum-dependent electronic structure and the polarization direction of laser pulses.^32^ This development provides a comprehensive explanation of the polarization-dependent amplitude of demagnetization in conventional magnets and effectively captures the emergence of asymmetric sublattice dynamics in altermagnets. This work provides a novel understanding of a-OISTR, clarifying the spin dynamics in 2D altermagnets with momentum-dependent electronic structures by laser control. The light-induced asymmetric spin canting and net magnetization suggest potential avenues for ultrafast spintronic applications. The capacity to optically generate and regulate a net magnetic moment on femtosecond time scales in altermagnets has the potential to facilitate the development of innovative memory and logic devices. For instance, the transient ferrimagnetic state induced by a-OISTR may serve as a platform for ultrafast spin-current generation and manipulation, facilitating the development of spin-wave emitters or detectors. The combination of these functionalities with the inherent high-frequency dynamics and stray-field immunity of altermagnets positions 2D altermagnets as a promising material class for next-generation spintronics technologies that require low power and high speed.

The alterations in the direction and magnitude of magnetic moments can be accurately measured and detected in experimental settings. The experimental probing of altermagnetic order is contingent on symmetry-sensitive responses. Angle-resolved photoemission spectroscopy directly resolved momentum-dependent spin splitting in epitaxial RuO_2_ films^36^ and *g*-wave bands in MnTe.^37,38^ Magneto-optical Kerr effect measurements detected time-reversal symmetry breaking in RuO_2_ ^34^ and Mn_5_Si_3_,^35^ while X-ray magnetic circular dichroism revealed sublattice-selective responses in α-MnTe.^39^ In addition, the exploration of layered AM, as prepared in experiments,^11,12^ will be expanded to include 2D AM. Furthermore, we simulated the off-axis dielectric tensor element ε_*xy*_ to describe the magneto-optical response of Fe_2_WTe_4_, as shown in Figure S11. We observed a significant difference of ε_*xy*_ between α = 0° and 90°, which confirms that the predicted spin reorientation could be detectable in time-resolved experiments, such as the time-resolved magneto-optical Kerr effect.^46^ This provides a potential means of observing spin reorientation. Therefore, experimental validation of our theoretical predictions of net spin moment and the transition from AM to the ferrimagnetic state could be achieved through the utilization of ultrafast spectroscopy techniques in the near future.

Our simulations resolve the early stage spin dynamics within the first 50 fs; however, the impact of electron–phonon coupling beyond this time scale remains unexplored. Further investigation is required to determine whether the net magnetic moment sustains growth over extended periods, with the potential to drive a phase transition to ferromagnetic order. It is imperative to elucidate the interplay between phonon excitations and spin relaxation in altermagnets, as this may dictate the stability and evolution of the non-equilibrium magnetic state.

In summary, our work reveals previously unexplored ultrafast magnetization dynamics in 2D AMs, a class of magnetic materials characterized by compensated spin structures and momentum-dependent spin splitting. Using rt-TDDFT, we demonstrate that laser excitation induces strong asymmetric demagnetization between the two Fe sublattices in semiconducting AM Fe_2_WTe_4_. This imbalance results in the formation of a metastable ferrimagnetic state with a net magnetization of approximately 0.3 μ_B_ per unit cell within 45 fs. This laser-induced ferrimagnetism originates from an anisotropic form of OISTR, driven by the momentum-resolved spin-split band structure inherent to *d*-wave AM. In addition to asymmetrical demagnetization, we identify polarization-dependent non-collinear spin dynamics that manifest as unequal spin canting angles between Fe atoms, further enriching the spin response of the 2D AM system. Crucially, both net magnetization and asymmetric spin canting dynamics can be precisely modulated by the in-plane polarization angle of the laser. Our results reveal a new class of light–matter interaction phenomena in AM, expanding the landscape of optically driven ultrafast spin dynamics beyond conventional magnets.

## Supplementary Material



## Notes

Methods and computational details, band structure at different *U* values, normalized Fe atom-resolved spin moment under different laser parameters, normalized Fe atom-resolved spin moment and magnetization density, differences in the time-resolved occupation function and change in the spin-resolved charges, spin-resolved band structure, spin canting angle at α = 45°, change in *x*, *y*, and *z* components of spin moment of Fe atoms, *x* and *y* components of spin moment of Fe atoms at α = 45°, *x*, *y*, and *z* components of spin moment of Fe atoms at α = 0° under the SOC = 0.1 and 0.5, change in the *z* component of spin moment of Fe atoms at α = 0° under the SOC = 0.1, 0.5, and 1, and off-axis dielectric tensor element at α = 0° and 90° (PDF)
