# Supplementary material for: Asymmetric Spin Canting and Demagnetization Dynamics Driven by Laser Fields in Two-Dimensional Altermagnets
Source: Nano Lett. 2026 Feb 9;26(6):1995–2001. doi: 10.1021/acs.nanolett.5c04244 (PMC12922177; doi:10.1021/acs.nanolett.5c04244)
Supplement: Supplementary file 1 [file nl5c04244_si_001.pdf]

Supplementary Information

Asymmetric Spin Canting and Demagnetization Dynamics Driven by Laser Fields in Two-Dimensional Altermagnets

Shuo Li [1], Ran Wang [1], Thomas Frauenheim [1,2], Zhaobo Zhou [3*], Junjie He [1,3*]

1 Institute for Advanced Study, Chengdu University, Chengdu 610106, China

2 School of Science, Constructor University, Bremen, 28759, Germany

3 Faculty of Science, Charles University, Prague 12843, Czech Republic

* Corresponding authors.

Email: *zhaoboz@natur.cuni.cz, junjie.he@natur.cuni.cz*

Methods and computational details

To identify the spin dynamics of Fe_2WTe_4 under the influence of ultrafast laser pulses, we performed real-time time-dependent density functional theory (rt-TDDFT) calculations. The time evolving state functions (ψ) were calculated by solving the time dependent Kohn-Sham (KS) equation as follows:

$$i\frac{\partial\psi_j(\mathbf{r},t)}{\partial t} = \left[\frac{1}{2}\left(-i\nabla + \frac{1}{c}\mathbf{A_{ext}}(t)\right)^2 + \upsilon_s(\mathbf{r},t) + \frac{1}{2c}\boldsymbol{\sigma}\cdot\mathbf{B_s}(\mathbf{r},t) + \frac{1}{4c^2}\boldsymbol{\sigma}\cdot(\nabla\upsilon_s(\mathbf{r},t)\times(-i\nabla))\right]\psi_j(\mathbf{r},t) ,$$

where $\mathbf{A_{ext}}(t)$ and σ represent vector potential and Pauli matrices. The KS effective potential $\upsilon_s(\mathbf{r},t) = \upsilon_{ext}(\mathbf{r},t) + \upsilon_H(\mathbf{r},t) + \upsilon_{xc}(\mathbf{r},t)$ can be decomposed into the external potential υ_{ext}, the classical Hartree potential υ_H, and the exchange-correlation (XC) potential υ_{xc}, respectively. The KS magnetic field can be written as $\mathbf{B_s}(\mathbf{r},t) = \mathbf{B_{ext}}(\mathbf{r},t) + \mathbf{B_{xc}}(\mathbf{r},t)$, where $\mathbf{B_{ext}}$ and $\mathbf{B_{xc}}$ represent the magnetic field of the applied laser pulse with an additional magnetic field and XC magnetic field, respectively. The last term in Eq. (1) stands for the SOC effect. The magnetization density can be calculated as $\boldsymbol{m}(\mathbf{r},t) = \sum_j \psi_j^*(\mathbf{r},t)\boldsymbol{\sigma}\psi_j(\mathbf{r},t)$ and the integral of this vector field over the unit cell leads to the spin angular momentum.

Calculations at ground state were performed at the density functional theory (DFT) level using the Vienna ab initio simulation package (VASP),[1,2] using the Perdew–Burke–Ernzerhof (PBE) exchange–correlation functional.[3] the Brillouin zone was represented by a Γ Monkhorst-Pack grids of 11 × 11 × 1 for structure relaxation and electronic structures of Fe_2WTe_4. A correction of U = 3 eV only for the electronic structures of Fe element is employed.[4] An energy cut-off of 500 eV was used to determine the self-consistent charge density for the plane wave basis sets. The structures were fully optimized until the maximum force on atoms was lower than 0.01 eV/Å and the total energy variation was lower than 1.0×10^{-6} eV. A vacuum of approximately 10 Å was added in the perpendicular direction to the slab model.

For rt-TDDFT simulations, we only considered the spin polarized electron dynamics based on the Born-Oppenheimer approximation. The electron-phonon coupling effects are not taken into account. Photoinduced dynamics calculations were made using a fully non-collinear version of rt-TDDFT and a full-potential augmented plane-wave ELK code.[5] A regular mesh in a k-space of 8 × 8 × 1, a smearing width of 0.027 eV, and a time step of Δt = 0.1 a.u. were used to simulate

excited dynamics. The laser pulses that were used in the present study were linearly polarized at a selected frequency. All calculations were performed using adiabatic local spin density approximations (ALSDA)[6] with the correction of U = 3 eV for Fe atoms.

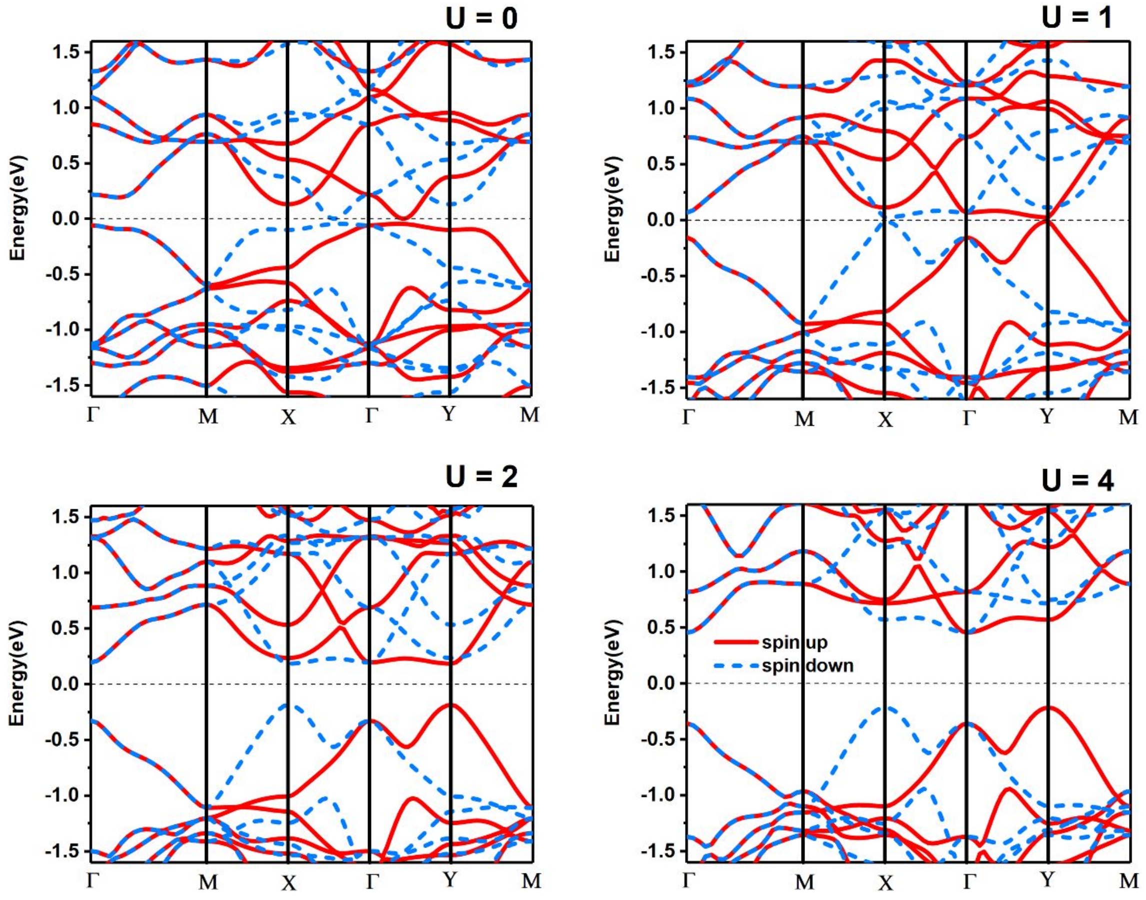

Figure S1 Band structure of Fe_2WTe_4 without SOC at different U values.

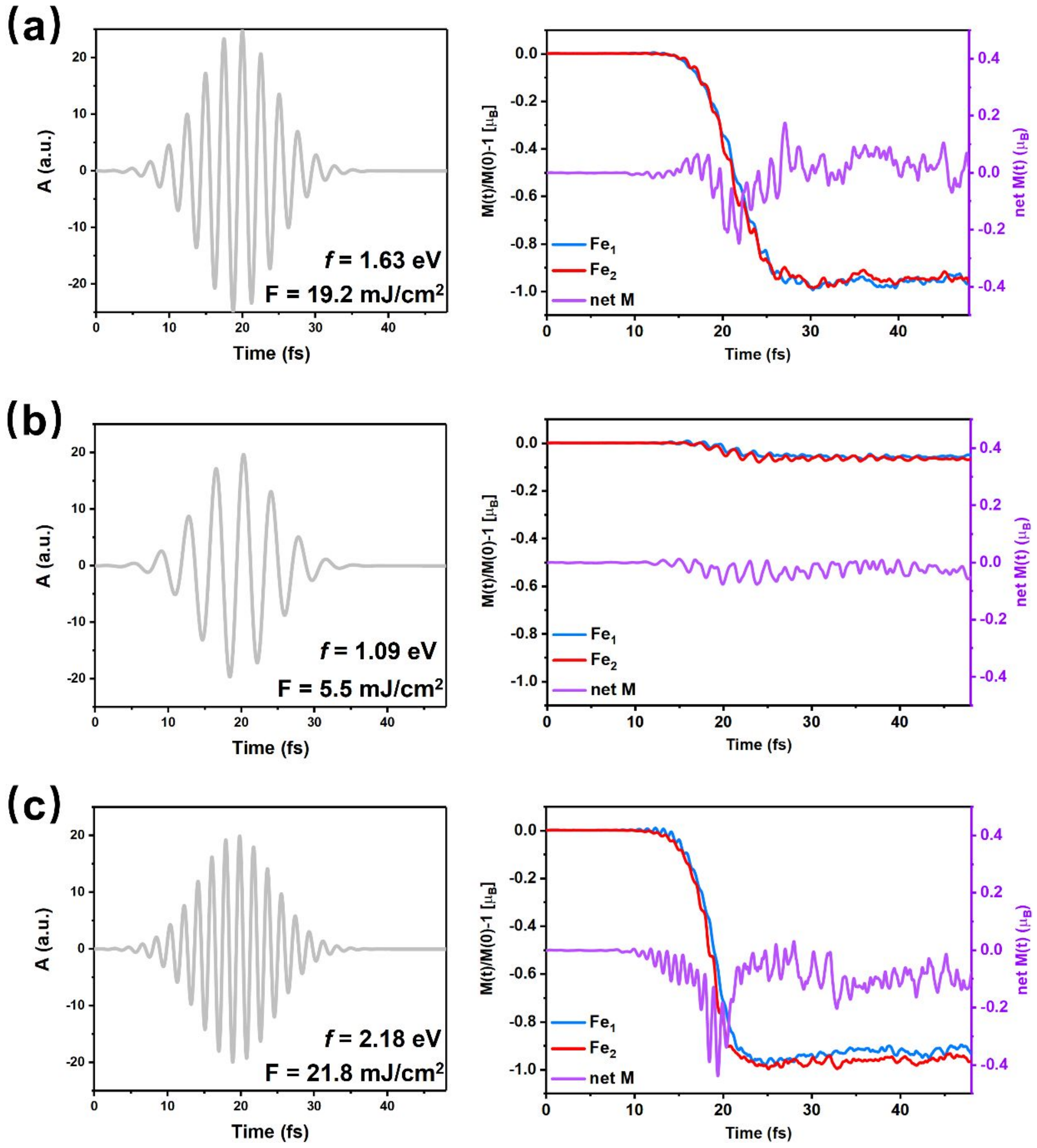

Figure S2 The vector potential of the laser pulse (a) the amplitude A = 25 a.u., the frequency f = 1.63 eV, and the fluence F = 19.2 mJ/cm^2. (b) the amplitude A = 20 a.u., the frequency f = 1.09 eV, and the fluence F = 5.5 mJ/cm^2. (c) the amplitude A=20 a.u., the frequency f = 2.18 eV, and the fluence F = 21.8 mJ/cm^2. The corresponding normalized Fe atom-resolved spin moment as a function of time at $\alpha = 0°$ on right panel. The net magnetic moment (net M) is shown in purple.

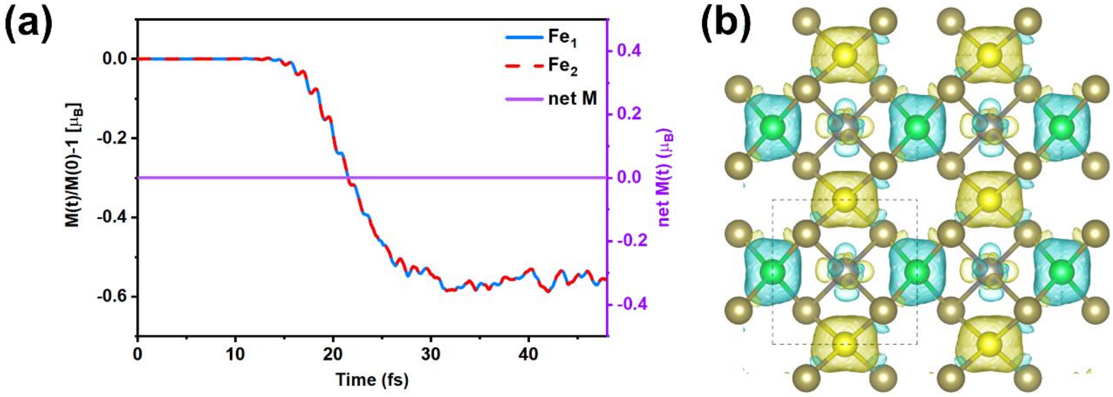

Figure S3 (a) Normalized Fe atom-resolved spin moment as a function of time at α = 45°. The net magnetic moment (net M) is shown in purple. (b) Magnetization density of Fe_2WTe_4 at α = 45°. Yellow and green domains indicate the spin-up and spin-down density, respectively. The isosurface is set to 0.0012 e/Bohr^3.

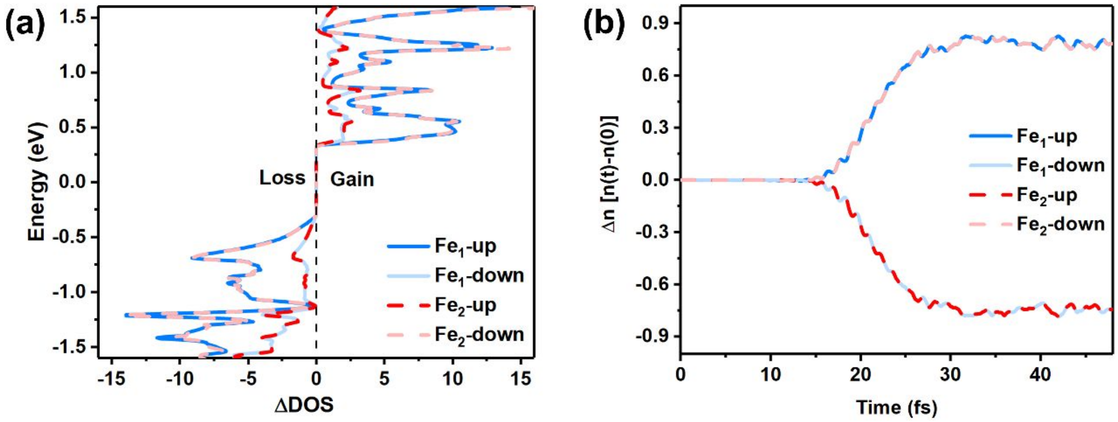

Figure S4 (a) Differences in the time-resolved occupation function ΔDOS(t) at t=43.5 fs, and at α = 45°. The negative value signifies a loss of electrons, and a positive value signifies a gain of electrons. (b) Change in the spin-resolved charges Δn of two Fe atoms at α = 45°. The positive/negative value represents the increase/decrease of charges.

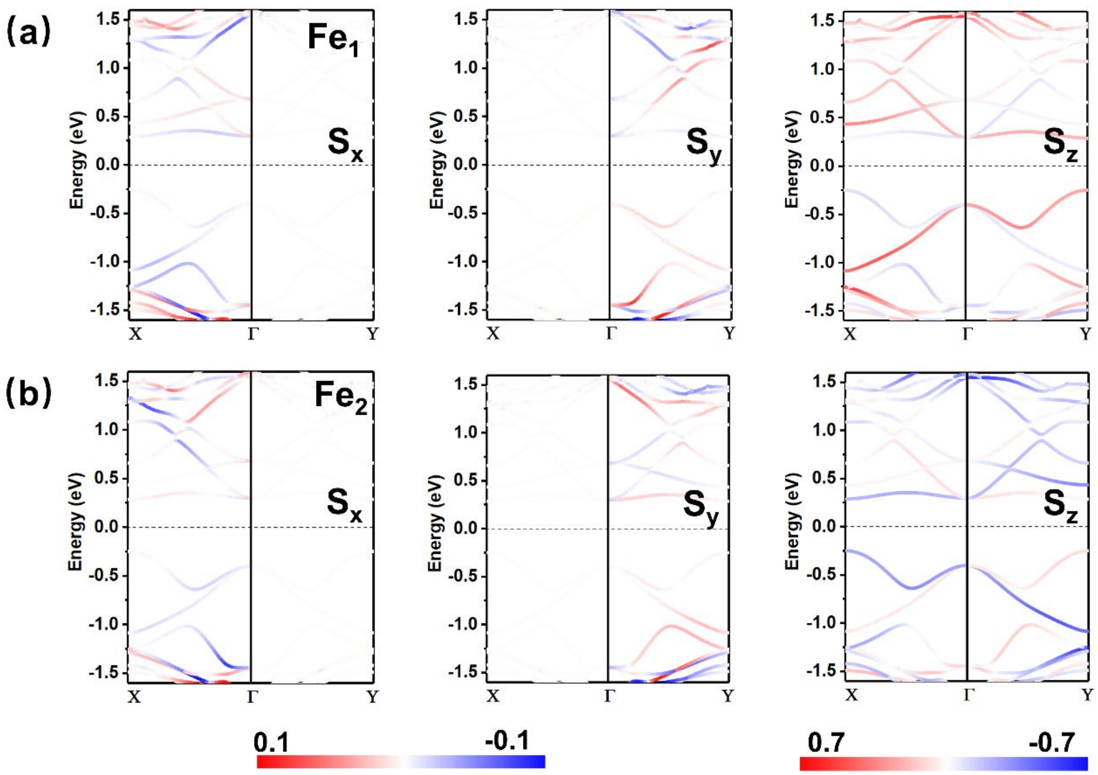

Figure S5 Spin-resolved band structure of Fe_2WTe_4 projected on (a) Fe_1 and (b) Fe_2 atoms. The three subpanels refer to S_x, S_y and S_z components of the spin density, the red (blue) color indicates a positive (negative) value. During these calculations, the magnetization is set perpendicular to the plane.

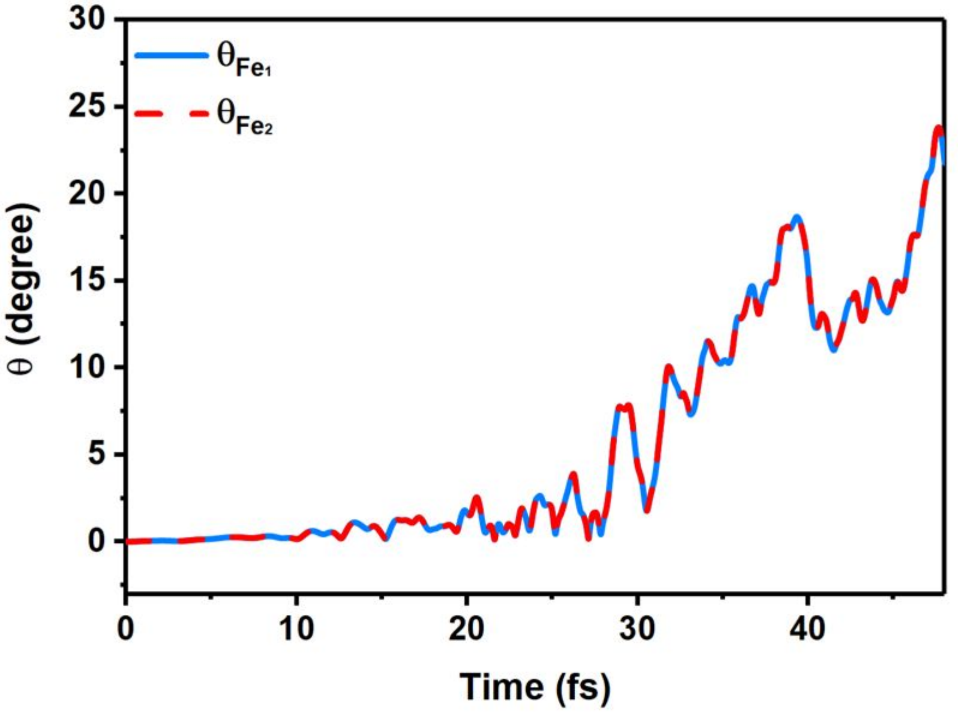

Figure S6 The spin canting angle θ as a function of time at α = 45°.

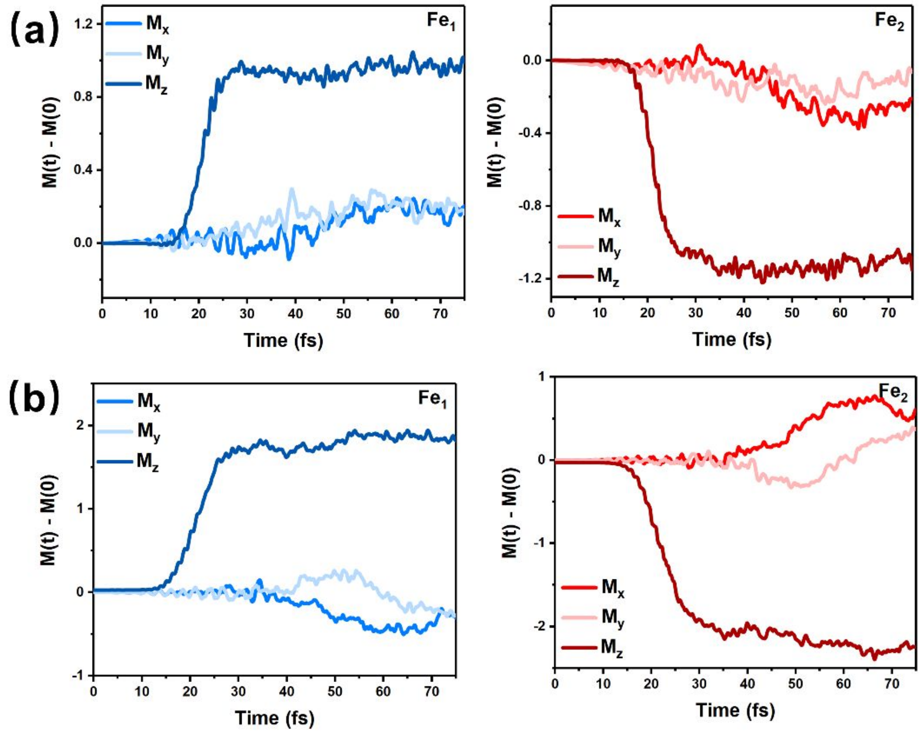

Figure S7 The change in x, y, and z component of spin moment of Fe atoms as a function of time (a) at α = 0° and (b) at α = 90°

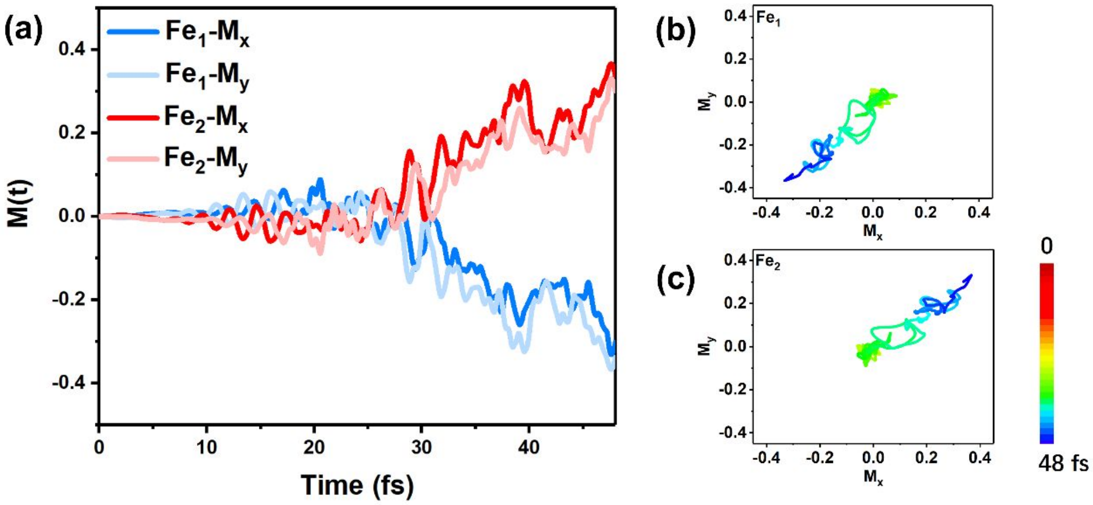

Figure S8 (a) The x and y component of spin moment of Fe atoms as a function of time at α = 45°. (b and c) The x and y component of spin moment of Fe atoms on the M_x-M_y plane as a function of time at α = 45°. The color bar serves as the timescale.

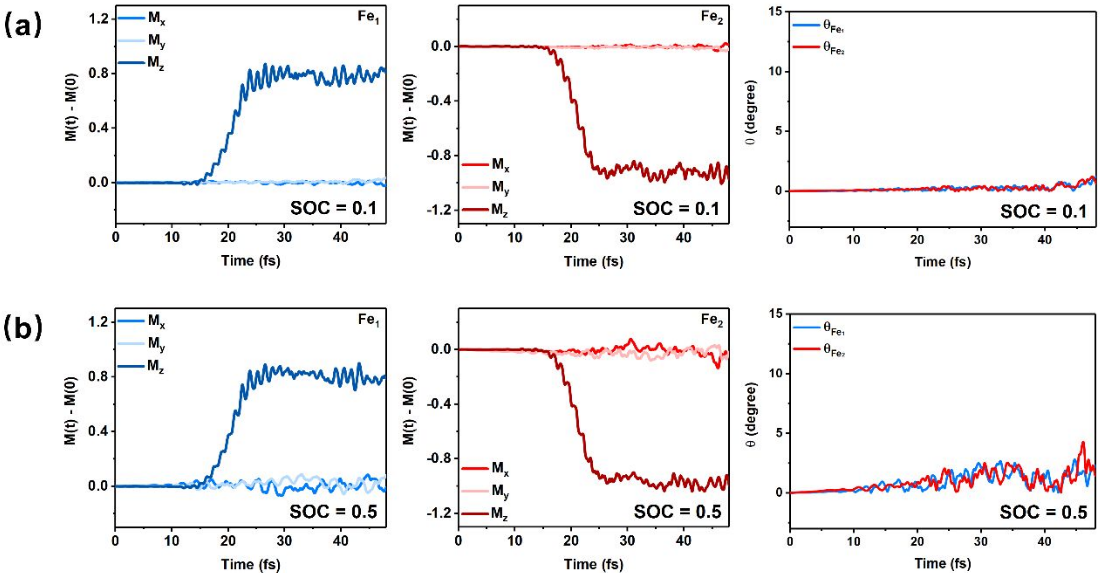

Figure S9 The change in x, y, and z component of spin moment of Fe atoms as a function of time (on left) and the spin canting angle θ as a function of time (on right) at α = 0° under the SOC scaled by factors of (a) 0.1 and (b) 0.5.

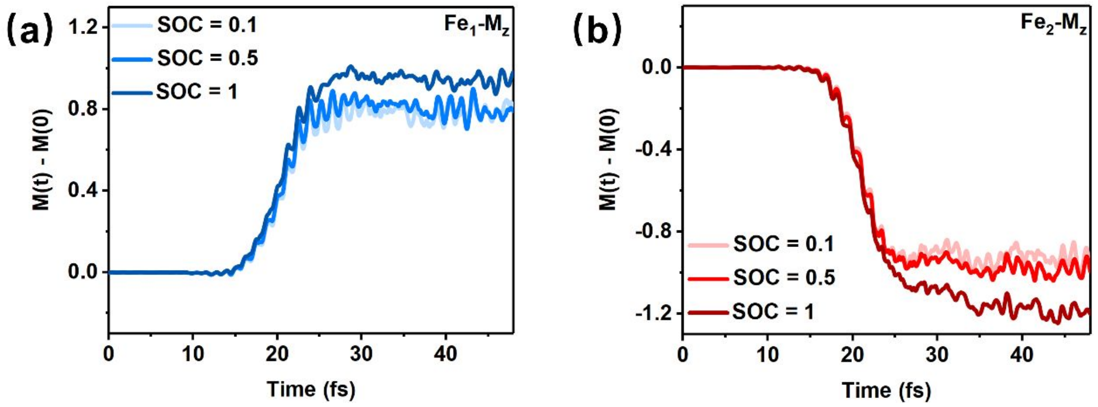

Figure S10 The change in z component of spin moment of Fe atoms as a function of time at α = 0° under the SOC scaled by factors of 0.1, 0.5, and 1.

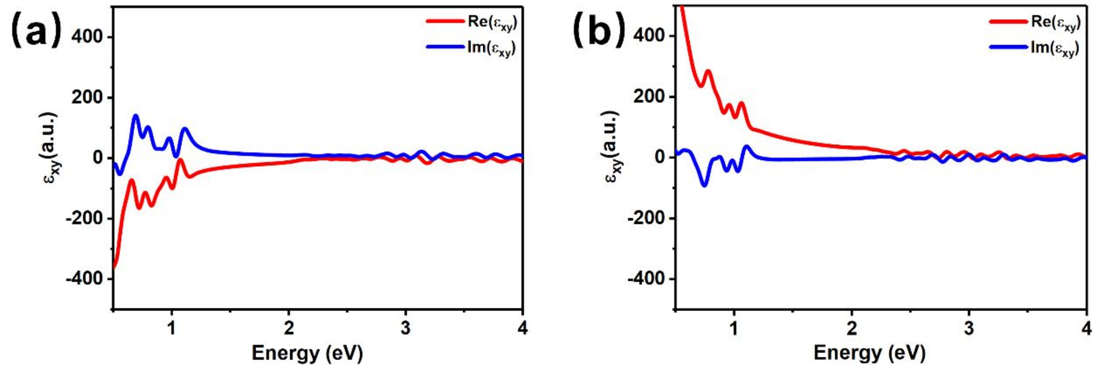

Figure S11 The off-axis dielectric tensor element ε_{xy} at (a) α = 0° and (b) α = 90°.

References

1. Kresse G, Hafner J. Ab initio. *Physical Review B* **47**, 558-561 (1993).
2. Kresse G, Joubert D. From ultrasoft pseudopotentials to the projector augmented-wave method. *Physical Review B* **59**, 1758-1775 (1999).
3. Perdew JP, Burke K, Ernzerhof M. Generalized Gradient Approximation Made Simple. *Physical Review Letters* **77**, 3865-3868 (1996).
4. Dudarev SL, Botton GA, Savrasov SY, Humphreys CJ, Sutton AP. Electron-energy-loss spectra and the structural stability of nickel oxide: An LSDA+U study. *Phys. Rev. B* **57**, 1505-1509 (1998).
5. Elk code, elk.sourceforge.net
6. Von Barth U, Hedin L. A local exchange-correlation potential for the spin polarized case. i. *J. Phys. C: Solid State Phys.* **5**, 1629 (1972).
